# Effect of Ki-67 proliferation index on survival in large cell neuroendocrine carcinoma of the lung: correspondence

**DOI:** 10.1590/1806-9282.20242090

**Published:** 2025-06-16

**Authors:** Yuanli Xia

**Affiliations:** 1 Zhejiang University, School of Medicine, The First Affiliated Hospital, The Clinical Research Center for Respiratory Diseases of Zhejiang Province, Thoracic Disease Center, Department of Respiratory Disease – Hangzhou, China.

Dear Editor,

We would like to share ideas on the publication "Effect of Ki-67 proliferation index on survival in large cell neuroendocrine carcinoma of the lung^1^". The article explored the potential relationship between the Ki-67 proliferation index and patient survival by retrospectively analyzing clinical and pathological data from 38 patients, providing important insights into this area of research. It was found that patients with a Ki-67 index ≥65% had a lower median overall survival than patients with a Ki-67 index <65%, and although the difference did not reach statistical significance, this trend echoed previous studies. The article's rigorous research methodology, including immunohistochemical quantitative Ki-67 index and Kaplan-Meier survival analysis, with meticulous data analysis and transparent results, lays a solid foundation for further research.

Despite the important academic value of the study, there are still some aspects worth improving and exploring. First, the sample size of the study was small; only 38 patients were included, of which women accounted for only 3%. The limitation of the sample may affect the statistical significance and the generalizability of the results, and the reliability of the results can be improved by multi-center cooperation or larger sample size studies in the future. Second, the potential clinical application of the Ki-67 index as a biomarker should be further explored; for example, whether clearer diagnostic criteria can be established and personalized treatment strategies can be designed for patients with a high Ki-67 index. In addition, for the finding of the relationship between right lung tumors and chronic diseases and survival, it is recommended that the biological mechanisms be further investigated to reveal potential tumor aggressiveness characteristics or therapeutic targets. We look forward to further validating its clinical value and exploring the possibility of personalized treatment through larger studies in the future.
